# Intranasal Delivery of Thermostable Subunit Vaccine for Cross-Reactive Mucosal and Systemic Antibody Responses Against SARS-CoV-2

**DOI:** 10.3389/fimmu.2022.858904

**Published:** 2022-05-03

**Authors:** Khue G. Nguyen, Siena M. Mantooth, Maura R. Vrabel, David A. Zaharoff

**Affiliations:** ^1^ Joint Department of Biomedical Engineering, University of North Carolina-Chapel Hill and North Carolina State University, Raleigh, NC, United States; ^2^ Comparative Medicine Institute, North Carolina State University, Raleigh, NC, United States

**Keywords:** Intranasal vaccination, COVID-19, mucosal immunity, SARS-CoV-2, Receptor binding domain (RBD), chitosan, CpG

## Abstract

Despite the remarkable efficacy of currently approved COVID-19 vaccines, there are several opportunities for continued vaccine development against SARS-CoV-2 and future lethal respiratory viruses. In particular, restricted vaccine access and hesitancy have limited immunization rates. In addition, current vaccines are unable to prevent breakthrough infections, leading to prolonged virus circulation. To improve access, a subunit vaccine with enhanced thermostability was designed to eliminate the need for an ultra-cold chain. The exclusion of infectious and genetic materials from this vaccine may also help reduce vaccine hesitancy. In an effort to prevent breakthrough infections, intranasal immunization to induce mucosal immunity was explored. A prototype vaccine comprised of receptor-binding domain (RBD) polypeptides formulated with additional immunoadjuvants in a chitosan (CS) solution induced high levels of RBD-specific antibodies in laboratory mice after 1 or 2 immunizations. Antibody responses were durable with high titers persisting for at least five months following subcutaneous vaccination. Serum anti-RBD antibodies contained both IgG1 and IgG2a isotypes suggesting that the vaccine induced a mixed Th1/Th2 response. RBD vaccination without CS formulation resulted in minimal anti-RBD responses. The addition of CpG oligonucleotides to the CS plus RBD vaccine formulation increased antibody titers more effectively than interleukin-12 (IL-12). Importantly, generated antibodies were cross-reactive against RBD mutants associated with SARS-CoV-2 variants of concern, including alpha, beta and delta variants, and inhibited binding of RBD to its cognate receptor angiotensin converting enzyme 2 (ACE2). With respect to stability, vaccines did not lose activity when stored at either room temperature (21-22°C) or 4°C for at least one month. When delivered intranasally, vaccines induced RBD-specific mucosal IgA antibodies, which may protect against breakthrough infections in the upper respiratory tract. Altogether, data indicate that the designed vaccine platform is versatile, adaptable and capable of overcoming key constraints of current COVID-19 vaccines.

## Introduction

As of March 2022, three vaccines have been fully approved or authorized for emergency use (EUS) by the U.S. FDA to prevent COVID-19. Outside of the U.S., an additional 10 vaccines have received full marketing approval with 19 more vaccines granted emergency used authorization. Given the rapidly evolving COVID-19 vaccine landscape, readers are encouraged to consult covid19.trackvaccines.org for up-to-date information. The remarkable efficacies of these vaccines and the unprecedented speed at which they have been developed are a testament to cutting-edge biomedical technologies and powerful academic-industry-government collaborations. It has been estimated that COVID-19 vaccines have prevented over one million deaths in the United States alone through November 2021 (1, 2). Nevertheless, significant opportunities for improvement exist in the areas of breakthrough infection prevention, vaccine stability, mass vaccination bottlenecks, and vaccine acceptance.

First, regarding breakthrough infections, although vaccinated individuals are 10 times less likely to be hospitalized, according to one CDC report (3), none of the current vaccines are capable of completely preventing SARS-CoV-2 infections. This lack of prevention is especially true for variants of concern. The CDC director, Dr. Rochelle Wolensky, has recently reported in an Associated Press interview that 75% of the first 40 U.S. confirmed cases of the Omicron variant were breakthrough infections in fully vaccinated individuals (4). Breakthrough infections, while generally not lethal, extend the circulation and transmissibility of SAR-CoV-2. Alternate routes of immunization have the potential to limit the frequency of breakthrough infections. Currently, all approved COVID-19 vaccines are administered as intramuscular (i.m.) injections. The i.m. route is very effective for generating antigen-specific IgGs in blood. However, i.m. immunization does not result in significant antibody responses in the upper respiratory tract where viruses are first encountered (5). In contrast, intranasal (i.n.) immunizations provide superior mucosal immunity and are more likely to prevent breakthrough infections at mucosal sites of virus entry (6).

Second, with respect to stability, mRNA-based vaccines require the most stringent storage conditions. CDC guidance states that Moderna’s elasomeran must be stored between -50°C and -15°C, while Pfizer/BioNTech’s tozinameran requires conditions between -90°C and -60°C. Once a multi-dose vial of either of these vaccines has been punctured, all doses must be used within 6 or 12 hours. Furthermore, once a multi-dose vial has been thawed, it cannot be refrozen. Other vaccines, including adenovirus-based vaccines from Johnson & Johnson (Ad26.COV2.S), Oxford-AstraZeneca (Vaxzevria), CanSinoBIO (Convidecia), and Gamaleya Research Institute (Sputnik V) require refrigeration temperatures between 2-8°C. Inactivated virus-based vaccines from Sinopharm (BBIBP-CorV) and Sinovac (CoronaVac) as well as Novovax’s subunit vaccine (NVX-CoV2373) also must be stored in a refrigerator. After being punctured, multi-dose vials must typically be used within 2 hours if kept at room temperature or 6 hours if stored between 2-8°C. These cold-chain or ultra-cold-chain requirements hinder vaccine access in rural communities and developing countries.

Third, the reliance on healthcare workers to administer i.m. vaccines to large populations creates a bottleneck that could be alleviated by exploring alternate routes and self-administration. Both microneedle patches, explored elsewhere (7), and i.n. dispensers can be leveraged for vaccine self-administration. Millions of people self-administer steroids and vasoconstrictors intranasally to prevent or treat allergic rhinitis. Although not without concerns, such as ensuring proper dosing, i.n. vaccine self-administration deserves consideration during emergent global pandemics. As an added benefit, neither microneedle patches nor i.n. vaccines generate biohazardous sharps (needles).

Fourth, concerning vaccine acceptance, despite the availability of free, highly effective COVID-19 vaccines, only 65.0% of the vaccine-eligible U.S. population is fully vaccinated as of March 2022 (8). Vaccine hesitancy is a complex issue with many roots. The novelty of the mRNA vaccine technology and the concern that these vaccines could alter the genome of an otherwise healthy individual are two pervasive, if not unfounded, concerns. Eliminating infectious and genetic material from a vaccine may enhance acceptance. Separately, for the estimated 10-20% of the population with trypanophobia, the development of a needleless strategy is essential for maximizing vaccination rates.

To overcome the above constraints of existing COVID-19 vaccines, updated design criteria were applied to engineer a novel vaccine. These criteria require that a new vaccine: (1) be stable at room temperature, (2) be effective *via* i.m. and i.n. routes, (3) be comprised of non-infectious and non-genetic material; and (4) be cross-protective against SARS-CoV-2 variants of concern. The resulting prototype vaccine platform consisted of the receptor binding domain (RBD) of the spike (S) protein, formulated in a chitosan (CS) solution. CS is a naturally occurring polysaccharide consisting of D-glucosamine and N-acetyl D-glucosamine units linked by β(1-4)glycosidic bonds. Previous research demonstrated that CS induced mixed Th1/Th2 responses against co-formulated antigens (9, 10). CS vaccine formulations were found to be non-toxic and biodegradable while reducing the dose of antigen needed for seroconversion (10). Furthermore, CS has been shown to have inherent immune-stimulating properties through cGAS-STING activation (11) and NLRP3 inflammasome induction (12–14). In clinical studies, CS solutions have been used in i.n. vaccines against influenza virus, *Corynebacterium diphtheriae* toxin, meningococcal group C, Norwalk virus, and human immunodeficiency virus 1 (15, 16). Altogether, CS has been shown, in multiple publications by multiple groups, to have potent adjuvant properties.

To evaluate prototype vaccines, initial preclinical studies focused on verifying the activity of CS/RBD formulations in laboratory mice following subcutaneous (s.c.) vaccination. The ability of immunoadjuvants CpG and IL-12 to boost and skew immune responses was explored. RBD mutants derived from SARS-CoV-2 variants of concern were used to probe cross-reactivity. Neutralization studies evaluated the inhibition of RBD-ACE2 binding by serum antibodies from vaccinated mice. To assess thermostability, CS/RBD vaccines were stored at room temperature (21-23°C) or 4°C for one month prior to immunization. Lastly, the induction of mucosal immunity following i.n. CS/RBD vaccination was investigated.

## Results

### Chitosan Formulation Enhances Antibody Responses to RBD Vaccinations

**Figure 1:**
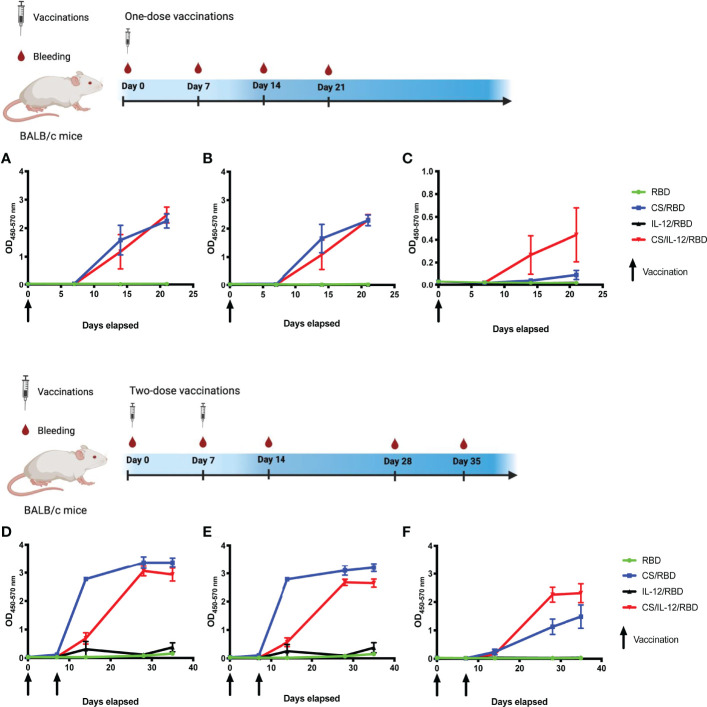
RBD-specific IgG antibody and isotype responses following s.c. vaccination. Mice (n=3 per group) were vaccinated once **(A–C)** or twice **(D–F)** with RBD alone, CS/RBD, IL-12/RBD or CS/IL-12/RBD. RBD-specific IgG **(A, D)**, IgG1 **(B, E)**, or IgG2a **(C, F)** levels in sera as a function of time after immunization were measured *via* ELISA. Vaccination schemas were created with BioRender.com.

Initial studies utilized the s.c. vaccination route to determine 1) if a CS formulation could enhance an RBD subunit vaccine, and 2) if the inclusion of additional adjuvants could skew the immune response towards a Th1 phenotype, which is believed to be necessary to prevent serious SARS-CoV-2 infections (17–19). In these studies, the cytokine interleukin-12 (IL-12) was used as the adjuvant due to its key role in driving Th1 immunity (20, 21). Mice were vaccinated either once or twice with unformulated RBD, CS/RBD, IL-12/RBD, or CS/IL-12/RBD. Following a single s.c. immunization with CS/RBD or CS/IL-12/RBD, significant levels of anti-RBD IgG were detected as soon as one week after vaccination (**Figure 1A**). RBD-specific antibody concentrations increased two weeks post-vaccination, even without a booster injection. Vaccination with RBD alone or IL-12/RBD did not result in detectable levels of anti-RBD IgG after a single vaccination (**Figure 1A**). These data suggest that RBD, like most subunit antigens, requires a vaccine delivery system to induce a productive adaptive immune response.

Booster vaccinations with CS/RBD or CS/IL-12/RBD markedly increased anti-RBD IgG production (**Figure 1D**). The anti-RBD response appeared to plateau two weeks after the second vaccination, implying that additional booster injections may not be necessary.

Antibody isotype studies were performed to determine the type of Th response generated by different RBD vaccines. CS/RBD resulted in high levels of both IgG1 and IgG2a which is indicative of a mixed Th1/Th2 response (**Figures 1B, C, E, F**). These data are in good agreement with previous CS adjuvanted vaccines which demonstrate mixed Th1/Th2 responses to other antigens (22). IL-12 appeared to skew the CS/RBD response toward a Th1 phenotype with lower levels of IgG1 and higher levels of IgG2a (**Figures 1B, C, E, F**).

### Chitosan and RBD Induce High Titer Antibodies With Strong Avidity

**Figure 2:**
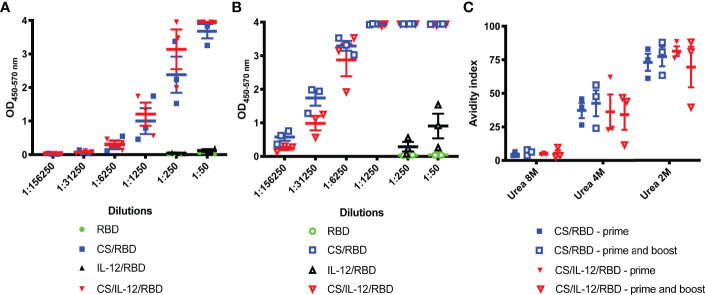
Serum RBD-specific antibody titers and avidity. Mice (n=3 per group) were vaccinated s.c. with RBD alone, CS/RBD, IL-12/RBD or CS/IL-12/RBD. Serum anti-RBD IgG levels in serial dilutions were measured 21 days after one **(A)** or two **(B)** vaccinations. Anti-RBD avidity index **(C)** was calculated as the ratio of IgG binding to RBD in the presence vs. the absence of urea.

A subsequent study set out to quantify antibody titers and avidity following s.c. immunization with CS-formulated vaccines. Serum samples two weeks post-vaccination displayed high titers of anti-RBD IgG among the CS/RBD and CS/IL-12/RBD groups after only a single vaccination (**Figure 2A**). Anti-RBD IgG could be detected at dilutions greater than 1:6250 for both CS/RBD and CS/IL-12/RBD vaccines. A second vaccination significantly improved antibody titers (**Figure 2B**). After the booster, anti-RBD antibodies could be detected in sera that was roughly 250 times more dilute (**Figure 2B**). When administered with CS, IL-12 did not seem to increase antibody titers compared to CS/RBD vaccinations (**Figures 2A, B**). However, in the absence of CS and with a much lower signal, IL-12 was found to increase anti-RBD titers modestly (**Figures 2A, B**). Taken together, these data demonstrate that IL-12 has a slight but positive effect on antibody production.

Avidity of anti-RBD IgGs was assessed *via* a urea dilution assay. In 2M urea, antibodies from cohorts receiving a CS-formulated vaccine were found to have similar high binding with avidity indices ranging from 39.6% to 88.2%. (**Figure 2C**). Surprisingly, booster vaccinations did not appear to increase avidity (**Figure 2C**). In 4M urea, avidity indices decreased, and all antigen-antibody binding was disrupted in 8M urea. Despite differences in antibody titers, there were no differences in antibody avidity among the vaccinated cohorts (**Figure 2C**).

### CpG Enhances Chitosan-RBD Antibody Production

**Figure 3:**
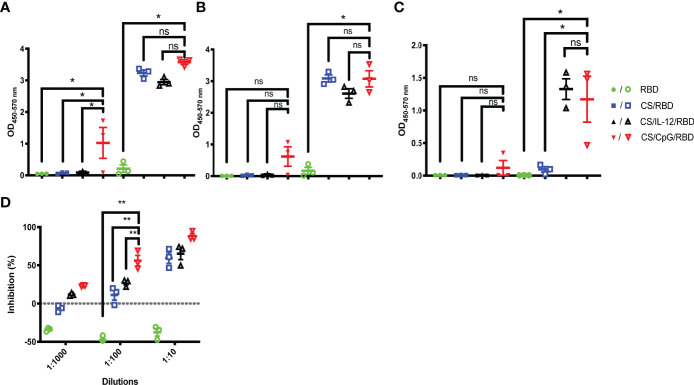
RBD-specific antibody and isotype responses comparing IL-12 and CpG as immunoadjuvants. Mice (n=3 per group) were vaccinated once (closed symbols) or twice (open symbols) with RBD alone, CS/RBD, CS/IL-12/RBD or CS/CpG/RBD. RBD-specific IgG **(A)** IgG1 **(B)** and IgG2a **(C)** levels in sera 21 days after the final vaccination were measured via ELISA. *p < 0.05 via one-way ANOVA with Tukey’s posttest, “ns” indicates “not significant”. The presence of neutralizing antibodies in sera of mice vaccinated twice were measured as the inhibition of RBD binding to ACE2 via a surrogate virus neutralization test (sVNT) **(D)**. **p < 0.05 via two-way ANOVA with Tukey’s posttest.

Given concerns over the translatability and long-term thermostability of IL-12 (23–26), the widely explored immunomodulator CpG was incorporated in vaccine formulations. Mice were vaccinated with one or two doses of unformulated RBD, CS/RBD, CS/IL-12/RBD, or CS/CpG/RBD. Inclusion of CpG yielded a measurable amount of anti-RBD IgG following just one vaccination (**Figure 3A**). In fact, anti-RBD IgG levels were significantly higher after a single CS/CpG/RBD vaccination than after RBD, CS/RBD, or CS/IL-12/RBD vaccination (**Figure 3A**). After booster vaccinations, no statistical differences in anti-RBD IgG concentrations were observed among the CS-formulated vaccines (**Figure 3A**).

With regard to IgG subtype responses, anti-RBD IgG1 and IgG2a concentrations were measurable after one vaccination only when CpG was included in the formulation. (**Figures 3B, C**). After two vaccinations, CS/RBD with and without an additional adjuvant yielded high levels of anti-RBD IgG1, but only the addition of either IL-12 or CpG yielded measurable levels of anti-RBD IgG2a (**Figures 3B, C**).

Using a SARS-CoV-2 surrogate virus neutralization test (sVNT), serum antibodies from the CS/CpG/RBD vaccinated animals were found to inhibit about 88.4% RBD binding to ACE2 at a 1:10 serum dilution (**Figure 3D**). As expected, neutralization decreased with increasing dilutions. However, the CS/CpG/RBD vaccine resulted in higher levels of neutralization compared to CS/IL-12/RBD and CS/RBD vaccines at all three dilutions levels (p<0.05 *via* two-way ANOVA with Tukey’s posttest) (**Figure 3D**).

### CS/CpG/RBD Vaccines Exhibit Cross-Reactivity Against Specific VOCs

**Figure 4:**
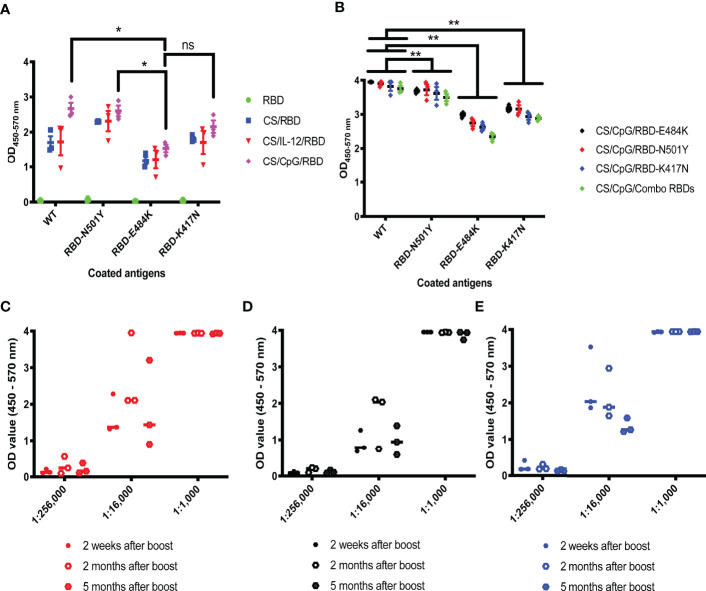
Cross-reactivity and durability of antibody responses to diverse CS/CpG/RBD vaccinations. Mice (n=3 per group) were vaccinated s.c. twice with RBD alone, CS/RBD, CS/IL-12/RBD or CS/CpG/RBD and bled 21 days later. IgG specificity against RBD, RBD-N501Y, RBD-E484K and RBD-K417N was measured *via* ELISA **(A)**. Mice (n=3 per group) were vaccinated s.c. twice with CS/CpG/RBD-N501Y, CS/CpG/RBD-E484K, CS/CpG/RBD-K417N or a CS/CpG formulation with RBD, RBD-N501Y, RBD-E484K and RBD-K417N combined (“combo”). IgG specificity against RBD, RBD-N501Y, RBD-E484K and RBD-K417N was measured *via* ELISA **(B)**. Mice (n=3 per group) were vaccinated s.c. twice with CS/CpG/RBD-N501Y **(C)**, CS/CpG/RBD-E484K **(D)**, or CS/CpG/RBD-K417N **(E)**. Durability of IgG responses were measured *via* ELISA plates coated with the vaccine antigen, i.e. RBD-N501Y **(C)**, RBD-E484K **(D)** or RBD-K417N **(E)**, using sera collected 2 weeks, 2 months and 5 months after booster vaccinations. *p < 0.05 *via* one-way ANOVA with Tukey’s posttest. **p < 0.05 *via* two-way ANOVA with Tukey’s posttest. ns, not significant.

To determine if antibodies generated by CS/CpG/RBD vaccines may be protective against SARS-CoV-2 variants of concern (27), three relevant RBD mutations, RBD-N501Y, RBD-K417N, and RBD-E484K, served as targets in customized ELISAs. Antibodies from mice vaccinated with CS/CpG/RBD recognized all three mutations, with comparable binding to wild-type RBD and RBD-N501Y and reduced binding to RBD-E484K and RBD-K417N (p<0.05 *via* two-way ANOVA with Tukey’s posttest) (**Figure 4A**). Interestingly, the CS/CpG/RBD vaccine consistently induced higher levels of anti-RBD IgG against all RBDs compared to the CS/IL-12/RBD vaccines, however, differences were not statistically significant.

Subsequent studies sought to determine whether RBD mutants could also serve as antigens in vaccine formulations to offer broad or adaptable protection. As above, mice were vaccinated following a prime-boost schedule with RBD-N501Y, RBD-K417N, or RBD-E484K, as well as a combination of the four RBDs, 3 mutants plus 1 wild-type, formulated with CS and CpG. Serum antibodies from all four cohorts displayed robust recognition of both wild-type RBD and RBD-N501Y, with no difference among the vaccine cohorts (**Figure 4B**). Binding slightly decreased across all vaccination groups when RBD-E484K and RBD-K417N served as ELISA targets (**Figure 4B**). These results suggest that the detection antigen used to coat ELISA plates had a greater influence on antibody binding than the vaccine antigen.

To assess the durability of vaccine responses, the mice were bled 2 and 5 months post-vaccination. Anti-RBD IgG antibody levels did not significantly change over the 5 month period (**Figures 4C–E**). Although it is difficult to correlate age-related data between mice and humans, 5 months represents about one-fifth of a mouse’s average lifespan.

### CS/CpG/RBD Vaccines Are Stable at Room Temperature

**Figure 5:**
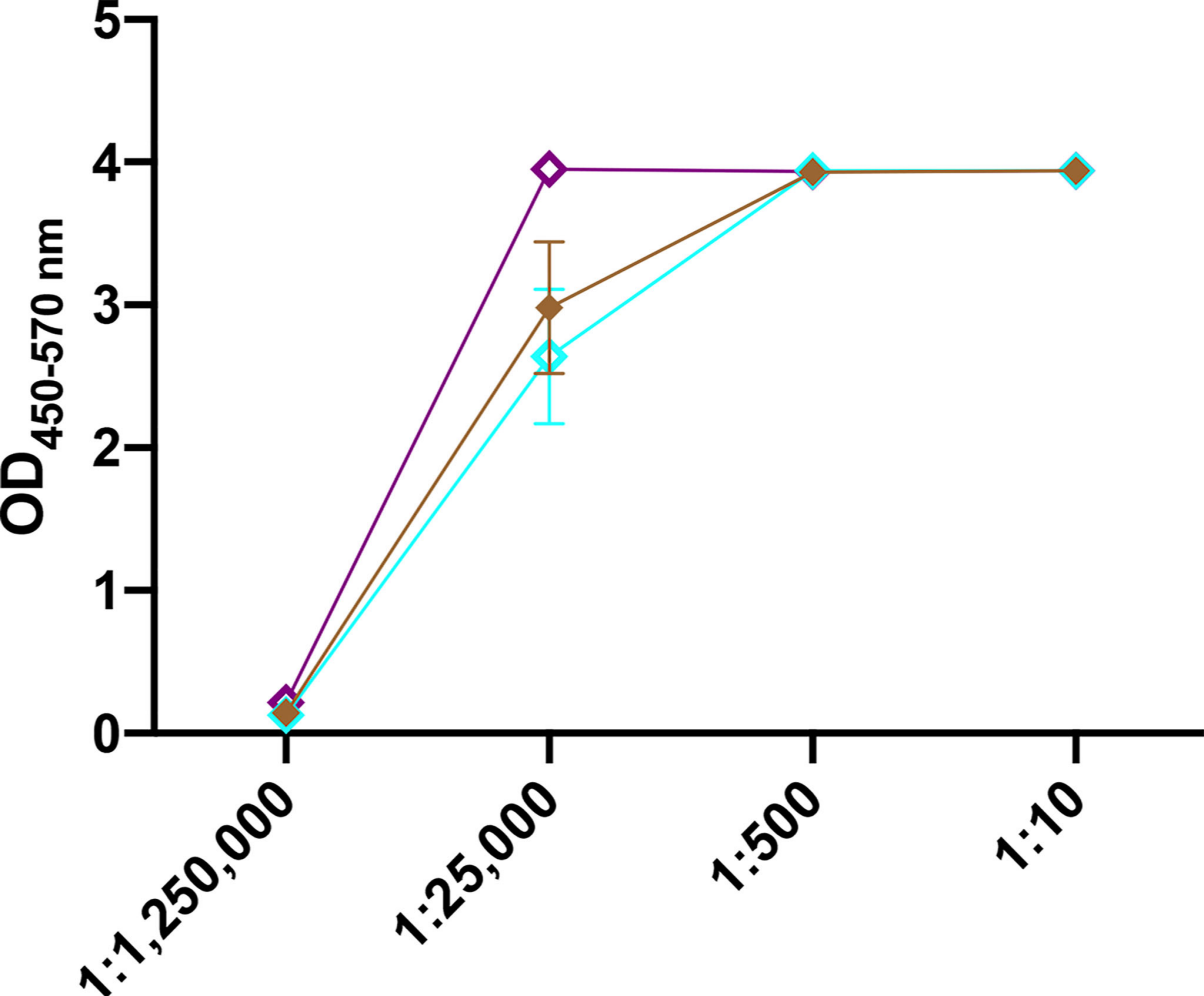
Effect of vaccine storage on efficacy. Mice (n=3 per group) were vaccinated s.c. twice with CS/CpG/RBD prepared fresh, stored at 4°C for 1 month or stored at room temperature (21-22°C) and bled 21 days later. RBD- specific IgG levels in serially diluted sera were measured *via* ELISA.

To gauge vaccine stability, responses to CS/CpG/RBD vaccines following a 1 month storage at either 4°C or at room temperature (21-23°C) were assessed. A freshly prepared vaccine was used for comparison. All vaccine cohorts developed strong anti-RBD IgG antibody responses (**Figure 5**). Interestingly, the vaccine stored at room temperature appeared to induce higher antibody levels than either the freshly prepared vaccine or the vaccine kept at 4°C at the 1:25,000 sera dilution (**Figure 5**). Antibody responses were nearly identical at all other dilutions.

### Intranasal CS/CpG/RBD Vaccination Induces Robust Mucosal IgA Responses

**Figure 6:**
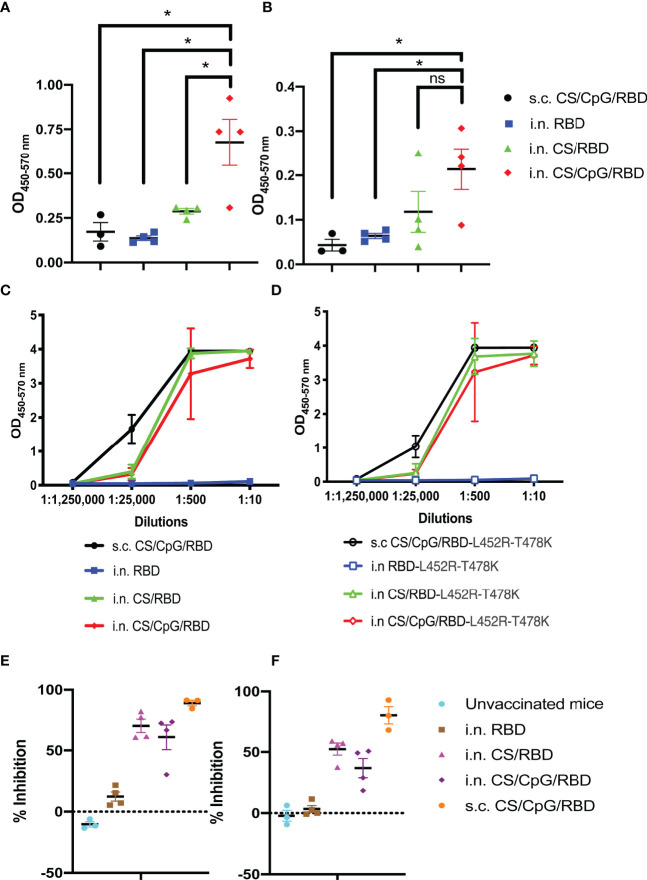
Intranasal vaccination with CS/CpG/RBD results in mucosal and systemic immunity. Mice (n=4 per group) were vaccinated twice i.n with RBD alone, CS/RBD or CS/CpG/RBD. For comparison, an additional cohort (n=3) was vaccinated twice s.c. with CS/CpG/RBD. RBD-specific IgA responses in sera **(A)** and nasal rinses **(B)** 14 days after booster immunizations were detected *via* ELISA. Binding of serum IgG to RBD **(C)** or RBD-L452R-T478K **(D)** was measured *via* ELISA. Neutralizing antibody responses were determined by inhibition of RBD **(E)** or RBD-L452R-T478K **(F)** binding to ACE2 by diluted serum samples. *p < 0.05 *via* one-way ANOVA with Tukey’s posttest. ns, not significant.

After validating the activity and stability of CS/CpG/RBD vaccines *via* s.c. injections, culminating experiments focused on generating mucosal immunity in the upper respiratory tract following i.n. vaccination. Mucosal immunity could help reduce viral spread among vaccinated individuals and also assist in preventing breakthrough infections. Mice were vaccinated i.n. with unformulated RBD, CS/RBD, or CS/CpG/RBD following a prime-boost schedule. A s.c. CS/CpG/RBD cohort was included as a control. Two weeks post-boost, sera from mice receiving i.n. CS/CpG/RBD contained the highest levels of anti-RBD IgA (p<0.05 *via* one-way ANOVA with Tukey’s posttest) (**Figure 6A**). Similarly, anti-RBD IgA levels in nasal washes were highest in mice vaccinated with i.n. CS/CpG/RBD (**Figure 6B**). In contrast, s.c. vaccination resulted in poor induction of anti-RBD IgA in both sera and nasal wash samples (**Figures 6A, B**). Furthermore, the importance of CS as a vaccine/delivery enhancer was underscored by the inability of unformulated i.n. RBD to induce significant anti-RBD IgA or IgG.

Serum IgGs from i.n. CS/RBD- or CS/CpG/RBD-vaccinated mice were found to recognize both wild-type RBD as well as RBD from the SARS-CoV-2 delta variant, RBD-L452R-T478K at comparable levels to s.c. CS/CpG/RBD-vaccinated mice (**Figures 6C, D**). As with the IgA analyses, the unadjuvanted i.n. RBD vaccine was found to be insufficient and did not result in IgG against either wild-type or delta variant RBD (**Figures 6C, D**). In neutralization assays, IgGs from s.c. CS/CpG/RBD-vaccinated mice demonstrated the highest level of inhibition, 89.1% and 80.5% against wild-type and RBD-L452R-T478K binding to ACE2, respectively (**Figures 6E, F**). Although i.n. vaccination with CS/RBD and CS/CpG/RBD resulted in slightly less inhibition of RBD to ACE2 binding, the data demonstrate that robust systemic immune responses can be achieved *via* the intranasal route.

## Discussion

The primary goal of this project was to develop an alternative COVID-19 vaccine that was capable of overcoming limitations of currently approved vaccines. Acquired data demonstrate that CS/CpG/RBD vaccines are stable at room temperature for at least one month and effective at inducing durable (**Figure 5**), high avidity (**Figure 2C**) RBD-specific neutralizing antibody responses following either s.c. (**Figure 3**) or i.n. (**Figure 6**) immunizations. To our knowledge, only two other COVID-19 vaccines, Oxford/AstraZeneca’s chimpanzee adenovirus vector-based ChAdOx1 nCOV-19 and a recombinant Newcastle Disease Virus-based vaccine by Laboratorio Avi-Mex, Mexico, have been developed for both i.m. and i.n. administration (28–30). Although mixed vaccination routes were not explored with the CS/RBD vaccine platform, it is an attractive option for future investigation given the success of heterologous prime/boost schemas (31, 32).

The induction of anti-RBD mucosal IgAs following i.n. vaccination is a key feature of the CS/CpG/RBD platform as this isotype is better suited to prevent breakthrough infections along upper respiratory surfaces than serum IgG, which can prevent serious infection and hospitalization (33–35). Although breakthrough infections in vaccinated individuals generally result in milder symptoms compared to infections in unvaccinated individuals (36), a vaccine capable of preventing breakthrough infections altogether would likely reduce the duration of viral circulation within a community.

The i.n. CS/CpG/RBD vaccine compared favorably with other i.n. COVID-19 vaccines in development. As of March 2022, at least 13 and 36 i.n. COVID-19 vaccine candidates were in clinical and pre-clinical testing, respectively (37). Data from several of these vaccines are available in peer-reviewed publications (34, 38–47). The Oxford/AstraZeneca ChAdOx1 nCoV-19 vaccine, when administered i.n. to rhesus macaques, induced high RBD-specific IgG and IgA titers in sera (34). In nasal swabs, RBD-specific IgA were detectable after a prime vaccination and increased significantly following a booster vaccination. Importantly, i.n. vaccination, but not i.m vaccination, resulted in reduced virus concentrations in nasal swabs following SARS-CoV-2 challenge in hamsters and macaques (34, 38, 39). Similar, IgA responses and durable protection from upper and lower respiratory tract infections were achieved following i.n. vaccination in mice (34) and macaques (42).

An adenovirus type 5-vectored vaccine encoding RBD (AdCOVID) was found to induce RBD-specific IgA in the respiratory tract and sera of i.n. vaccinated mice (40, 41). Although effective in protecting mice from a lethal SARS-CoV-2 challenge, development of AdCOVID by Altimmune has been discontinued due to lower than expected immune responses in a Phase I trial. A single i.n. dose of a replication-competent chimeric bovine/human parainfluenza virus type 3 (B/HPIV3) expressing pre-fusion stabilized (S-2P) SARS-CoV-2 S spike protein (B/HPIV3/S-2P) in hamsters, resulted in robust serum IgA and IgG responses to the SARS-CoV-2 S protein (40). SARS-CoV-2 could not be detected in the nasal tissues of vaccinated animals (40).

Overall, i.n. COVID-19 vaccines have been dominated by virus-based vectors. Viral vectors are attractive as they can be engineered to incorporate genes encoding diverse antigens and result in high transfection efficiency. However, viral vectors can be limited by scalability issues, safety concerns, heterogeneous antigen expression and pre-existing immunity (43). Subunit vaccines are generally safer although poorly immunogenic. Several very recent publications support subunit-based i.n. COVID-19 vaccination through formulation with different adjuvants (44, 48). Among those conferring mucosal immunity, a single i.n. vaccination with a spike protein adsorbed to liposomes encapsulating a STING agonist was found to induce mucosal IgA along the respiratory tracts of mice (43). Another unique vaccine, comprised of a stabilized spike protein fused to outer membrane vesicles (OMVs) from Neisseria meningitidis, provided protective mucosal IgA which administered i.n., but not i.m. to hamsters (44). Another group found that when RBD was encapsulated in trimethyl chitosan nanoparticles, i.n. delivery to mice resulted in RBD-specific IgA responses in lungs (46).

Given the constant threat of SARS-CoV-2 mutation and the development of vaccine-evading variants (49), it was important to show that antibodies generated by CS/CpG/RBD were capable of binding RBD mutants from major variants including Alpha (RBD-N501Y), Beta (RBD-K417N,E484K,N501Y) and Delta (RBD-L452R-T478K) (**Figures 4**, **6**). It was equally important to demonstrate that multiple RBD mutants could be formulated with the CS/CpG platform with no loss of activity (**Figure 4**). These findings underscore the broad protection yet flexibility of the CS/CpG/RBD platform for seasonal or yearly updates.

Regarding vaccine formulation, the inclusion of CS was found to be essential as vaccination with RBD alone was ineffective (**Figure 1**). CS was previously found to stimulate antigen-specific immune responses through diverse mechanisms including prolonged antigen retention (22, 50), STING activation (11) and NLRP3 inflammasome activation (12). The inclusion of CpG boosted anti-RBD titers and promoted IgG1 to IgG2a subtype switching (**Figure 3**). The adjuvant mechanisms of CpG through are well-studied and reviewed elsewhere (51–54). The combination of CS and CpG have been used previously in formulations of multiple vaccine candidates, demonstrating their effectiveness in enhancing immune responses (55).

Lastly, the use of non-infectious, non-genetic material may help with vaccine acceptance. As of March 2022, only 65% of vaccine-eligible individuals in the U.S. have been fully vaccinated (8). Given the widespread availability of three incredibly efficacious vaccines, the inability to achieve higher vaccination rates despite high reveals a complex challenge that is complicated by a great deal of distrust and/or misinformation among U.S. citizens. Excluding infectious and genetic material from vaccine formulations may alleviate lingering concerns over intentional or unintentional genetic manipulation (56, 57). Furthermore, subunit-based vaccines have a long track record of success with minimal adverse events (58). This alone is not likely to inspire a 100% vaccination rate. However, any improvement in vaccine acceptance could help save lives.

Furthermore, improved vaccine delivery logistics may also help with vaccine uptake. For instance, i.n. vaccinations avoid creation of biohazardous sharps and could eliminate the aforementioned healthcare worker bottleneck. In fact, given the likely need for periodic booster vaccinations (59–61), delivery of pre-loaded, thermostable i.n. vaccines *via* mail for self-administration becomes increasingly attractive. In the U.S., vaccination rates peaked at 499,013,558 individuals per day in December 2021 (62). This peak rate was limited by the availability of qualified healthcare workers to administer vaccines rather than demand for vaccine or vaccine supply. A vaccine-by-mail strategy has no such logistical limits.

There are several limitations of the current study which also reveal opportunities for continued investigation. First and foremost, no virus challenge studies were performed. CS/CpG/RBD vaccines were found to induce antibodies that block RBD-ACE2 binding, a critical step in SARS-CoV-2 infection. However, inhibiting RBD-ACE2 interaction *in vitro* does not guarantee protection from SARS-CoV-2 infection *in vivo*. A second limitation of the current study is the lack of comparison to existing mRNA vaccines due to sourcing issues. Given their tremendous success, mRNA vaccines can and should be used as a benchmark for novel COVID-19 vaccines. Planned studies will compare CS/CpG/RBD and mRNA vaccines directly in terms of anti-RBD titers, anti-RBD IgA production and thermostability. Lastly, the thermostability studies are limited in terms of duration and temperature as the CS/CpG/RBD vaccines were not expected to maintain 100% effectiveness after storing at room temperature for 1 month. Future studies will incorporate longer and warmer storage conditions to validate the potential to use this vaccine in environments lacking access to continuous refrigeration.

Despite the above limitations, CS/CpG/RBD vaccines demonstrate promise and appear to address several concerns hindering current COVID-19 vaccines. In addition to tackling the ongoing pandemic, the versatile CS/CpG/RBD platform is well-suited to combat the spread of future respiratory virus outbreaks through adaptations in antigen targets and vaccination route.

## Materials and Methods

### Animals

Female BALB/c mice, 8 to 12 weeks of age, were purchased from Charles River Laboratories (Wilmington, MA). Animal use strictly adhered to the Public Health Service Policy on Human Care and Use of Laboratory Animals. All experiments involving laboratory animals were approved by the Institutional Animal Care and Use Committee at North Carolina State University. Every effort was taken to reduce, refine and replace animal testing where possible.

### Vaccine Formulations

Wild-type (Z03483) and mutant (Z03533, Z03535, Z03536, Z03613) recombinant SARS-CoV-2 RBD polypeptides were purchased from Genscript (Piscataway, NJ). Chitosan acetate (30 – 200 kDa, 80–90% deacetylated) was obtained from Heppe Medical Chitosan GmbH (Halle, Germany). Recombinant mouse IL-12 (mIL-12) was synthesized and purified in-house as described previously (63–66). Class B CpG oligonucleotides (CpG-B 2006) were a generous gift from Checkmate Pharmaceuticals (Cambridge, MA). Dulbecco’s modified phosphate buffered saline (DPBS, 21040CV) was purchased from Corning.

Each s.c. vaccine dose contained 5μg RBD ± 10μg CpG ± 1μg IL-12 mixed in 1.5% (w/v) chitosan acetate solution in DPBS. S.c. vaccines were administered as a single 50μl injection in a shaved flank. Each i.n. vaccine dose contained 10μg RBD ± 10μg CpG in 1.0% (w/v) chitosan acetate solution in DPBS, for an overall volume of 40μL. For vaccination preparation with CpG, chitosan acetate was first dissolved in DPBS at a concentration of 18.8 (i.n.) or 21.1 (s.c.) mg/mL. Subsequently, RBD was added to the chitosan solution. CpG was diluted to a 1 mg/mL stock solution, and 10μg CpG from this stock was added to the vaccine formulation as a final step. I.n. vaccines were administered to isoflurane-anesthetized mice in four increments. First, 10μL of the vaccine was pipetted dropwise directly under the left nostril, allowing the mouse to inhale between each placed droplet. Approximately 1-2 minutes later, 10μL was delivered to the right nostril. Each mouse was recovered for 30-45 minutes before the procedure was repeated. All vaccines were formulated immediately prior to use with the exception of the stability studies in which vaccines were formulated and stored as indicated.

### Vaccination Study Design

Mice were vaccinated 1 or 2 times as indicated. Blood was collected *via* mandibular/facial vein bleeding at specific time points after treatment (67). Whole blood was allowed to clot for 1 to 2 hrs at room temperature before centrifugation at 2,000 x g for 15 minutes to collect sera. Nasal washes were collected after euthanasia by exposing the trachea and inserting a 24G i.v. catheter (Terumo). 200μL of cold DPBS was pushed, *via* syringe, into the trachea, through the nasal cavity and collected from the nostrils. Sera or nasal washes were stored at -80°C after collections. Samples were thawed in 4°C overnight before performing assays.

### Anti-RBD Antibody Detection

Anti-RBD antibodies in sera were detected by enzyme-linked immunosorbent assay (ELISA) as described previously (34, 40, 44). Briefly, high binding 96-well Maxisorp plates (Thermo Scientific) were coated overnight at 4°C with 1 µg/mL of an RBD target in PBS. Plates were rinsed three times with PBS and 0.05% Tween-20 (J62844, Alfa Aesar) then blocked for 1 hour at room temperature with 1% bovine serum albumin (BSA) in PBS (Thermo Scientific 37525). Plates were rinsed three times before addition of diluted sera or nasal washes and incubated overnight at 4°C. After rinsing three times as above, anti-mouse IgG (0107-05, SouthernBiotech), anti-mouse IgG1 (1071-05, SouthernBiotech), anti-mouse IgG2a (1081-05, SouthernBiotech), or anti-mouse IgA (1165-05, SouthernBiotech) conjugated with horseradish peroxidase (HRP) were added to plates and incubated for 1 hour at room temperature. Colorimetric signals were developed with 3, 3′, 5, 5′ - tetramethylbenzidine (TMB) (T0440, Sigma) and reactions were stopped with 2N HCl. Antibody levels were quantified *via* absorbance readings at 450 nm and 570 nm on a microplate reader (Cytation 5; Biotek, Winooski, VT).

### Avidity Assays

Various chaotropic agents disrupt immunological complexes produced by a certain antigen and a corresponding antibody in different ways. It was known that urea disrupted hydrogen bonding and Van der Waals forces (68). Therefore, we used urea (VWR 97063-804) as a chaotrope in an avidity ELISA based assay to determine the avidity of antibodies generated against RBD (68, 69). High-binding 96-well plates were coated, rinsed, blocked and rinsed as above. Diluted sera were incubated in coated plates for 1 hour at room temperature before adding different concentrations of urea (8M, 4M, 2M) at room temperature with vigorous shaking for 15 minutes. Anti-mouse IgG-HRP were then added for 1 hour at room temperature. Plates were developed with TMB and absorbance quantified on a microplate reader as above. The avidity index was calculated as the ratio of absorbances between urea-treated and untreated wells (69).

### Neutralization Assays SARS-CoV-2 Surrogate Virus Neutralization Tests (sVNT)

Neutralizing antibodies against SARS-CoV-2 RBD were detected using the SARS-CoV-2 surrogate virus neutralization test (sVNT) kit (L00847-A; Genscript) (70, 71). Briefly, RBD-HRP (provided by the kit) was incubated with serum samples and controls for 30 minutes at 37°C to allow for antibody binding. RBD-HRP/serum mixtures were then added plates pre-coated with human angiotensin-converting enzyme 2 (ACE2) for 15 minutes at 37°C. After rinsing three times with 1X Wash solution (provided by the kit), a TMB solution was added to quantify non-neutralized RBD-HRP that bound to ACE2. The reaction was stopped and read on a microplate reader as above. Absorbance was inversely related to the concentration of anti-SARS-CoV-2 neutralizing antibodies.

### Statistical Analysis

Differences in total antibody or neutralizing antibody levels generated by RBD, RBD/CS, RBD/CS/IL-12, or RBD/CS/CpG vaccinations were compared *via* one-way or two-way ANOVA with Tukey’s posttest. Statistical significance was accepted at the p ≤ 0.05 level. All analyses were conducted using GraphPad Prism 9 software (GraphPad Software, CA).

## Data Availability Statement

The original contributions presented in the study are included in the article/supplementary material. Further inquiries can be directed to the corresponding author.

## Ethics Statement

The animal study was reviewed and approved by North Carolina State University IACUC.

## Author Contributions

KN, SM, and DZ designed experiments, analyzed data, and wrote the manuscript. KN, SM and MV collected data. All authors edited and approved the manuscript. 

## Funding

This project was funded in part by a grant from the North Carolina Biotechnology Center (2020-FLG-3865) and two Graduate Research Fellowships from the National Science Foundation (SM and MV).

## Conflict of Interest

DZ is a consultant for Checkmate Pharmaceuticals.

The remaining authors declare that the research was conducted in the absence of any commercial or financial relationships that could be construed as a potential conflict of interest.

## Publisher’s Note

All claims expressed in this article are solely those of the authors and do not necessarily represent those of their affiliated organizations, or those of the publisher, the editors and the reviewers. Any product that may be evaluated in this article, or claim that may be made by its manufacturer, is not guaranteed or endorsed by the publisher.
